# Salivary Albumin and Alkaline Phosphatase in Infants: Exploring the Link Between Early Dental Development and Biomarkers

**DOI:** 10.1055/s-0045-1812106

**Published:** 2025-10-22

**Authors:** Sindy Cornelia Nelwan, Udijanto Tedjosasongko, Tania Saskianti, Ardianti Maartrina Dewi, Erika Setyowati, Sofia Tandya Putri, Nunthawan Nowwarote

**Affiliations:** 1 Department of Pediatric Dentistry, Faculty of Dental Medicine, Airlangga University, Surabaya, Indonesia; 2 Research Center for Biomaterial and Tissue Engineering, Airlangga University, Surabaya, Indonesia; 3 Department of Physiopathology and Oral Molecular, Centre de Recherche des Cordeliers, Universite Paris Cite, Sorbonne Universite, Paris, France

**Keywords:** tooth eruption, dental age, albumin, alkaline phosphatase, saliva, good health and well-being

## Abstract

**Objectives:**

This study aims to investigate the relationship between dental age and the levels of albumin and alkaline phosphatase (ALP) in saliva among children aged 6 to 24 months. This study evaluates their potential as noninvasive biomarkers for monitoring dental development. Specifically, it sought to determine whether these salivary proteins increase proportionally with dental age, providing an objective method to assess tooth eruption patterns in early childhood.

**Materials and Methods:**

Ethical approval was granted by the Airlangga University Hospital ethics committee to conduct this cross-sectional study in 33 children aged 6 to 24 months to collect data on dental age, albumin, and ALP levels in saliva. Saliva was taken using an absorbent paper and tested by enzyme-linked immunosorbent assay.

**Statistical Analysis:**

The data obtained were then analyzed by statistical tests using SPSS. Correlation test was analyzed using the Pearson's correlation test. A significance level of *p* < 0.05 was used to determine statistical significance.

**Results:**

The correlation test showed a significant relationship between dental age, albumin, and ALP levels in saliva ( *p* < 0.05), albumin and ALP levels in saliva increase along the children's dental age.

**Conclusion:**

This significant relationship suggests albumin and ALP in saliva as potential biomarkers in detecting dental age and tooth eruption in children.

## Introduction

Tooth eruption is a complex physiological process whereby teeth migrate from their intraosseous position within the jaws to their functional location in the oral cavity. The eruption of primary teeth typically begins around 6 months of age and is usually complete by 24 months. 1 The timing of tooth eruption is a frequent concern among parents and serves as a significant indicator of a child's overall growth and development. 2 Deviations from typical eruption patterns may reflect local or systemic abnormalities, making the monitoring of dental eruption clinically important. However, in practice, tooth eruption frequently occurs at ages that differ from established norms. 3

Accurately predicting the timing of tooth eruption or dental age poses challenges for both physicians and dentists. Although eruption charts provide general guidance, the variability among individuals is considerable due to a range of genetic, environmental, nutritional, and systemic influences. 4 For example, while primary incisors typically emerge between 6 and 12 months, this timeline can shift significantly depending on factors such as hereditary traits, malnutrition, or underlying medical conditions. 5 Misinterpretation of eruption timelines may lead to unnecessary parental anxiety, inappropriate referrals, or the oversight of serious developmental concerns.

Diagnostic limitations also hinder the assessment of eruption timing. Dental radiographs, while informative, pose unique challenges in infants and young children. Concerns primarily center on the risks associated with exposure to ionizing radiation. Despite technological advancements that have reduced radiation doses, infants are particularly susceptible due to their smaller size and rapidly developing tissues. 6 Repeated exposures—even at low doses—can cumulatively increase the risk of cellular damage. Moreover, obtaining clear images is often difficult due to poor patient cooperation, increasing the likelihood of motion artifacts and necessitating repeated imaging. In line with the ALARA (As Low As Reasonably Achievable) principle, imaging should be performed only when absolutely essential, with a preference for noninvasive alternatives when feasible. 7

Recent research has explored the utility of saliva as a noninvasive biomarker for monitoring dental development. Saliva, easily collected and rich in biological markers, has shown promise in reflecting both oral and systemic health conditions. 8 During the developmental period, changes in the salivary environment correspond with dental maturation. Salivary albumin, a predominant plasma protein, reflects mucosal permeability and plasma leakage into the oral cavity. 9 Alkaline phosphatase (ALP), another salivary biomarker, is associated with bone metabolism and has been proposed as an indicator of growth and developmental stages. 10 Alterations in these salivary proteins have been linked to phases of tooth eruption. Therefore, characterizing salivary protein profiles at various developmental stages may offer a valuable tool for early detection of eruption anomalies. Despite its potential, the literature on salivary biomarkers associated with tooth eruption remains limited, warranting further investigation.

## Materials and Methods

### Samples

This cross-sectional study was conducted using salivary samples collected from children aged 6 to 24 months. This age range was selected because it corresponds to the period during which the eruption of primary teeth begins and is generally completed. Ethical approval was obtained from the Ethics Committee of Airlangga University Hospital, Surabaya, Indonesia. Subjects were selected from patients visiting the hospital's pediatric clinic. Prior to participation, the study was explained to the parents or guardians, who provided written informed consent. Data on the children's general information, medical history, medication use, and growth and developmental status were obtained through parental interviews and medical records. The inclusion criteria comprised healthy infants and children aged 6 to 24 months with no known medical conditions, whose parents or guardians consented to their participation. Exclusion criteria included a history of acute or chronic systemic illness, recent or ongoing medication use, the presence of dental caries, periodontal disease, or gingival inflammation, as well as food or fluid intake within 1 hour prior to saliva collection. 11 This study employed purposive sampling with a convenience recruitment method, resulting in the enrollment of 33 participants. Sample collection was conducted during September 2024. After obtaining informed consent, demographic data recording, dental age assessment, and saliva sampling were performed concurrently in the morning between 8:00 and 10:00 a.m. This time frame was standardized to minimize potential variations in salivary composition related to circadian rhythm.

### Dental Age Analysis

**Fig. 1:**
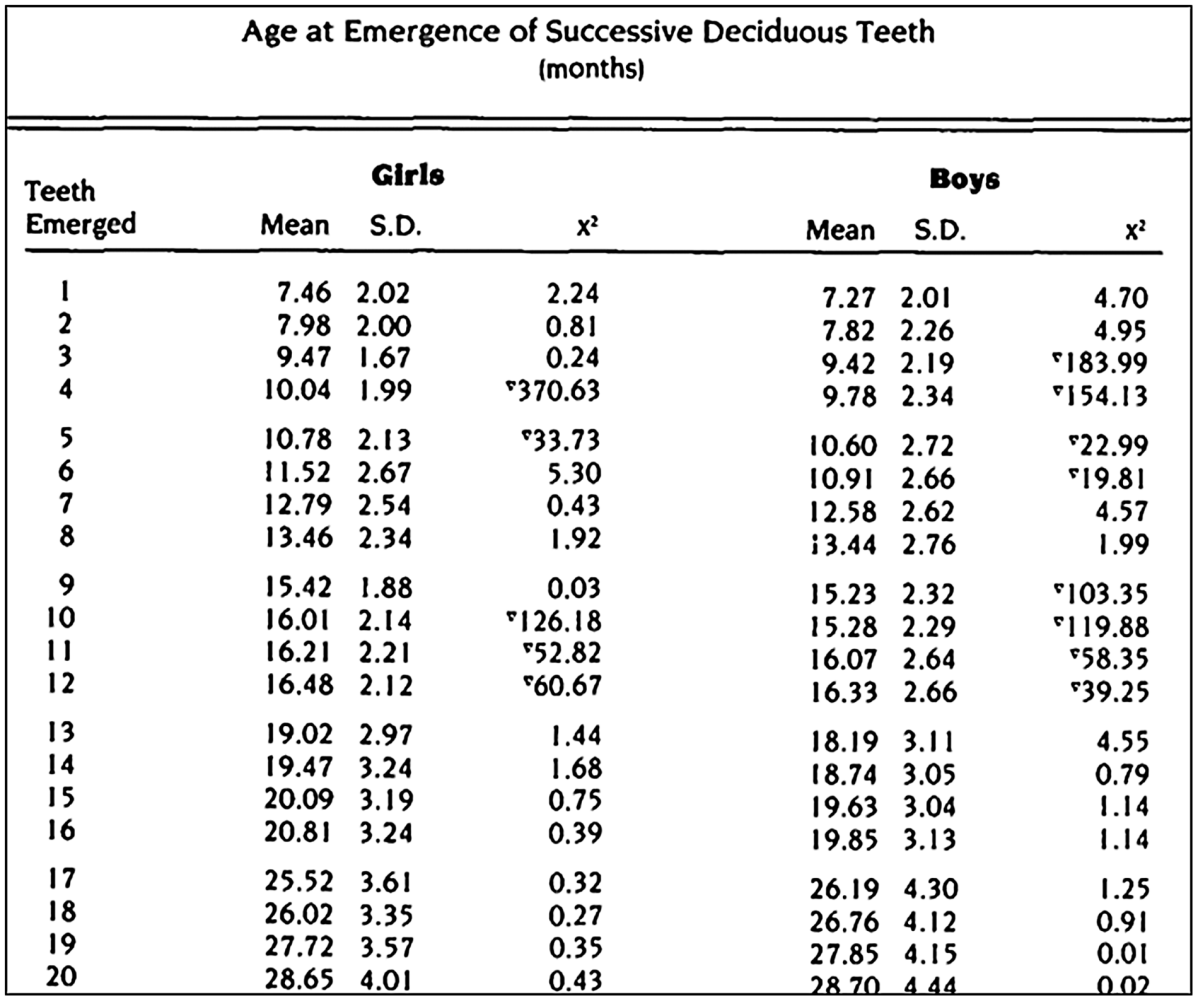
Dental age by Hagg–Taranger's method.

Dental age is widely regarded as a reliable indicator of both tooth eruption and biological maturity, as it offers a systematic and quantifiable framework for evaluating developmental progress. Compared with other skeletal markers, dental development is relatively less affected by environmental factors such as nutrition, thereby serving as a more stable measure of biological age. To obtain dental age data in this study, clinical examinations were conducted by a pediatric dentist utilizing a dental mirror and a focused light source, without the use of radiographic imaging. The examiner recorded the presence of erupted and erupting teeth on a standardized questionnaire and determined dental age based on the reference table provided by Hägg and Taranger method ( Fig. 1 ). 12 13 14 15

The Hägg and Taranger approach estimates dental age by simply counting erupted teeth and matching that count to sex-specific reference tables derived from a long-term Swedish longitudinal cohort followed from birth to 18 years. In practice, the physician counts the number of primary (and/or permanent) teeth that have erupted intraorally. Using the available reference curves and tables, this tooth count is then translated into an estimated dental age (with related variability). The method's precision is relatively tight for the primary dentition period (about ± 4 months at the 95% level), but it expands in the permanent dentition. The original work also documented sex differences—minimal in early primary emergence but favoring girls through much of the permanent dentition—and supplied standard deviation scores that allow expression of an individual's dental development relative to population norms. Recent methodological reviews and summaries continue to cite the Hägg–Taranger tooth-count framework as a practical clinical option for early childhood age estimation where radiography is undesirable. 15

### Saliva Collection and Enzyme-Linked Immunosorbent Assay Analysis

First, saliva collection was performed between 8 and 10 a.m. to minimize circadian variation. Participants refrained from eating or drinking for 1 hour prior to sampling to ensure unstimulated saliva. Unstimulated samples were obtained using PerioPaper strips placed in the oral cavity with sterile, disposable forceps for approximately 5 seconds, then immediately immersed in 0.5 mL phosphate-buffered saline. Following the collection, parents were provided with oral hygiene instructions. Each sample was transferred into a coded microcentrifuge tube and maintained at 0°C in a cooler box to prevent proteolysis. To reduce observer bias, each saliva sample was labeled only with a coded identifier at the time of collection. The laboratory staff responsible for analyzing salivary albumin and ALP levels were therefore unaware of the participant's age group, gender, or dental age classification. Sample decoding was performed only after the biochemical analyses were completed and results recorded.

Once the target sample size was reached, all tubes were transported to the Institute of Tropical Disease at Airlangga University and stored at −20°C until analysis. In this study, enzyme-linked immunosorbent assay (ELISA) was employed to quantify salivary albumin and ALP concentrations. ELISA was selected because it is a highly sensitive and specific method capable of detecting low concentrations of proteins and enzymes in small saliva samples, which is essential in pediatric populations where sample volumes are limited. ELISA was performed using commercially available kits for albumin and ALP (BT Laboratory), following the manufacturer's protocols for reagent preparation, standard dilution, and sample handling. After completing the assay procedures, absorbance was measured at 450 nm using a microplate reader. Albumin and ALP concentrations were subsequently determined based on the standard curves generated from the absorbance data. 16

### Data Analysis

Statistical analyses were performed using SPSS software (version XX; IBM Corp., Armonk, New York, United States). Data normality was assessed with the Shapiro–Wilk test, and homogeneity of variance was evaluated using Levene's test. For normally distributed data, correlations were examined using the Pearson's correlation coefficient. A significance threshold of *p* < 0.01 was applied. In addition to the overall analysis, the data were further stratified into three pediatric age groups (6–12, > 12–18, and > 18–24 months) and by gender. This stratification was undertaken to capture developmental changes that may be masked within broader age categories and to assess potential sex-related differences in salivary protein and enzyme expression during early childhood, as previously reported in the literature.

## Results

A total of 33 saliva samples were analyzed, consisting of 17 males and 16 females. The mean chronological age of participants was 14.51 ± 4.82 months (range: 6–24 months), while the mean dental age was 11.98 ± 5.82 months (range: 6–28.65 months). The mean salivary albumin concentration was 28.44 ± 22.33 IU/L, and the mean ALP concentration was 584.52 ± 297.89 IU/L ( Table 1 ).

**Table 1:** Descriptive statistics of overall chronological age, dental age, and salivary biomarkers ( *N* = 33)

Variable	*N*	Mean ± SD	Median (range)
Chronological age (mo)	33	14.51 ± 4.82	15.0 (6–24)
Dental age (mo)	33	11.98 ± 5.82	10.91 (6–28.65)
Albumin concentration (IU/L)	33	28.44 ± 22.33	20.38 (9.55–130.75)
ALP concentration (IU/L)	33	584.52 ± 297.89	590.31 (109.43–1037.64)

Abbreviations: ALP, alkaline phosphatase; SD, standard deviation.

When stratified by age and gender ( Table 2 ), the male subgroup included 3 participants aged 6 to 12 months, 8 participants aged > 12 to 18 months, and 6 participants aged > 18 to 24 months. Female participants included 6 in the 6- to 12-month group, 6 in the > 12- to 18-month group, and 4 in the > 18- to 24-month group.

**Table 2:** Dental age and salivary biomarker concentrations by age group and gender

Gender	Age group (mo)	*N*	Variable	Minimum	Maximum	Mean ± SD
Male	6–12	3	Dental age (mo)	6.00	9.42	7.75 ± 1.21
			Albumin (IU/L)	13.45	17.55	15.95 ± 2.05
			ALP (IU/L)	205.40	359.50	307.65 ± 79.36
	> 12–18	8	Dental age (mo)	6.00	19.85	13.00 ± 4.38
			Albumin (IU/L)	14.52	54.09	31.05 ± 12.79
			ALP (IU/L)	208.16	951.46	703.75 ± 243.68
	> 18–24	6	Dental age (mo)	6.00	26.76	12.64 ± 7.61
			Albumin (IU/L)	16.13	57.68	27.57 ± 15.62
			ALP (IU/L)	269.34	1001.37	613.10 ± 286.35
Female	6–12	6	Dental age (mo)	6.00	10.04	8.35 ± 1.46
			Albumin (IU/L)	11.81	20.38	17.49 ± 3.19
			ALP (IU/L)	109.43	498.26	346.12 ± 152.33
	> 12–18	6	Dental age (mo)	6.00	16.48	11.72 ± 4.21
			Albumin (IU/L)	9.55	34.96	22.21 ± 9.69
			ALP (IU/L)	197.61	884.52	642.96 ± 263.02
	> 18–24	4	Dental age (mo)	6.00	28.65	17.99 ± 9.98
			Albumin (IU/L)	13.63	130.75	59.70 ± 52.22
			ALP (IU/L)	243.72	1037.64	780.86 ± 326.99

Abbreviations: ALP, alkaline phosphatase; SD, standard deviation.

**Fig. 2:**
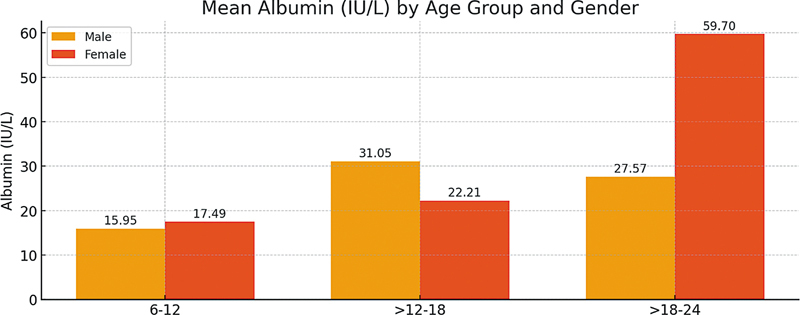
Mean salivary albumin levels by age group and gender.

**Fig. 3:**
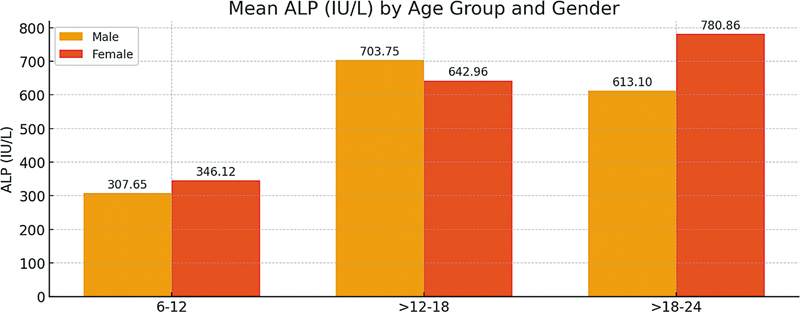
Mean salivary alkaline phosphatase (ALP) levels by age group and gender.

In males, dental age increased from 7.75 ± 1.21 months in the 6- to 12-month group to 13.00 ± 4.38 months in the > 12- to 18-month group, and then slightly decreased to 12.64 ± 7.61 months in the > 18- to 24-month group. Male salivary albumin levels rose from 15.95 ± 2.05 IU/L in the 6- to 12-month group to 31.05 ± 12.79 IU/L in the > 12- to 18-month group, before decreasing to 27.57 ± 15.62 IU/L in the > 18- to 24-month group. Male ALP levels also showed an increase with age, from 307.65 ± 79.36 IU/L in the youngest group to 703.75 ± 243.68 IU/L in the > 12- to 18-month group, with a subsequent decline to 613.10 ± 286.35 IU/L in the > 18- to 24-month group ( Figs. 2 and 3 ).

In females, dental age progressed more consistently across groups, from 8.35 ± 1.46 months in the 6- to 12-month group to 11.72 ± 4.21 months in the > 12- to 18-month group, and 17.99 ± 9.98 months in the > 18- to 24-month group. Albumin levels showed a steady increase across the age groups, from 17.49 ± 3.19 IU/L in the youngest group to 22.21 ± 9.69 IU/L in the > 12- to 18-month group, and 59.70 ± 52.22 IU/L in the > 18- to 24-month group. Similarly, female ALP concentrations rose with age, from 346.12 ± 152.33 IU/L in the 6- to 12-month group to 642.96 ± 263.02 IU/L in the > 12- to 18-month group, and further to 780.86 ± 326.99 IU/L in the > 18- to 24-month group ( Figs. 2 and 3 ).

Correlation analysis demonstrated significant positive associations between chronological age and dental age ( *r* = 0.595, *p* < 0.01), as well as with salivary albumin ( *r* = 0.538, *p* < 0.01) and ALP ( *r* = 0.576, *p* < 0.01). Dental age correlated strongly with both albumin ( *r* = 0.889, *p* < 0.01) and ALP ( *r* = 0.894, *p* < 0.01). Dental age demonstrated stronger coefficient correlations with salivary biomarkers than chronological age with the same salivary biomarkers ( Table 3 ).

**Table 3:** Pearson's correlation coefficients among chronological age, dental age, and salivary biomarkers

Variable	Chronological age	Dental age	Albumin concentration	ALP concentration
Chronological age	1	0.595 a	0.538 a	0.576 a
Dental age	0.595 a	1	0.889 a	0.894 a
Albumin concentration	0.538 a	0.889 a	1	–
ALP concentration	0.576 a	0.894 a	–	1

Abbreviation: ALP, alkaline phosphatase.

a Correlation is significant at the 0.01 level (two-tailed).

## Discussion

Predicting tooth eruption time is essential for ensuring proper dental and overall health, as it aids in diagnosing potential developmental issues, planning orthodontic treatments, and preventing future oral complications. Accurate knowledge of eruption patterns allows dentists and pediatricians to monitor a child's growth and detect abnormalities, such as delayed or premature eruption, which can indicate underlying medical conditions or jaw discrepancies. Timely prediction also supports the planning of interventions. Technique and tools in detecting tooth eruption must be noninvasive and safe considering its use should be done in children.

### Correlation Between Dental Age and Chronological Age

This study consistently observed that dental age lagged behind chronological age. This discrepancy has also been reported in other pediatric populations, and may reflect ethnic or population-specific eruption timing, nutritional influences, or environmental conditions. Previous studies have suggested that Asian children often present slightly delayed dental eruption compared with European cohorts, potentially due to differences in growth patterns and nutritional exposures. 4 Additionally, micronutrient deficiencies such as vitamin D insufficiency and overall growth retardation in early childhood have been implicated as contributors to delayed dental development. 17 Additionally, prematurity is another important factor; premature infants often experience delayed tooth eruption relative to their chronological age, which can be attributed to metabolic and nutritional disorders associated with early birth. This delay tends to be less pronounced when age is corrected for expected delivery date, pointing to the biological immaturity at birth as a cause. 18 Thus, the finding that dental age was lower than chronological age in this Indonesian cohort may reflect broader population-specific eruption trends and warrants further exploration in larger, multiethnic samples.

### The Relationship Between Albumin Levels in Saliva and Dental Age

The relationship between albumin and the eruption of primary teeth has been reported in several studies. For instance, Ruhl et al conducted a longitudinal study monitoring the overall protein composition and expression in infant saliva to hypothesize that specific protein expressions change during development and may correlate with tooth eruption. Their findings showed that overall saliva protein and glycoprotein patterns remained stable during infancy, except for mucin and albumin protein expression. Albumin, a marker of serum leakage, began to increase in saliva before tooth eruption. It shifted to significantly higher concentrations about a month before the first tooth erupted. This may result from increased epithelial permeability covering erupting teeth or microlesions caused by increased infant chewing activity. 19 Similarly, Morzel et al followed 73 infants longitudinally at ages 3 and 6 months. Saliva proteins were separated using sodium dodecyl sulfate-polyacrylamide gel electrophoresis electrophoresis and semiquantified through image analysis. Their study revealed that tooth eruption led to increased albumin concentrations. Albumin, derived from serum, enters the oral cavity through mucosal permeability or the gingival sulcus. 8

Salivary albumin levels have also been studied in relation to the mixed and permanent dentition phases. One study reported changes in albumin concentrations during tooth eruption. The highest albumin concentration was found in subjects with the most teeth, while the lowest concentration was recorded in edentulous children and adult. 20 Another study examining gingival crevicular fluid (GCF) samples from 26 healthy children aged 5 to 13 found that albumin increased in GCF during tooth eruption compared with teeth already erupted and in occlusal equilibrium. Tooth eruption is considered a physiological inflammatory condition, and albumin levels in GCF reflect an inflammatory response. 21

The above studies suggest tooth eruption may be associated with increased albumin levels in saliva. Albumin in saliva is a marker of serum leakage into the oral cavity. The increase in serum albumin is due to increased permeability of the inflamed epithelium or from gingival sulcus formation and a higher proportion of GCF in the overall saliva. In the phase of tooth eruption into the oral cavity, reduced enamel epithelium surrounds the crown of the tooth until eruption and immediately before eruption, this dental epithelial layer and the cells of the basal layer of oral epithelium fuse and migrate into the connective tissue. As the tooth penetrates the oral mucosa to enter the oral cavity, the reduced enamel epithelium develops into junctional epithelium, a nonkeratinized epithelium that begins to cover the incisal areas of the enamel, forming a continuum with the oral epithelium, while the reduced enamel epithelium and ameloblasts still cover the more apical parts of the enamel. This process of junctional epithelium formation continues and a space, called the gingival sulcus, forms between the enamel and the free gingiva. This gingival sulcus is the estuary of GCF which contains albumin, GCF then becomes part of saliva. 22

### The Relationship Between Alkaline Phosphatase in Saliva and Dental Age

Salivary ALP is one of the biomarkers often used to identify growth stages. ALP levels increase significantly during growth spurts in infancy and puberty, then decrease in adulthood. Previous research has shown that there is a relationship between salivary ALP levels and dental age as well as chronological age in children during the mixed dentition period. 23 The correlation among these three aspects can be used for diagnostic purposes. This study confirms that a similar correlation exists during the phase of primary tooth eruption. The more teeth that have erupted, the higher the ALP levels in the saliva. Conversely, if fewer teeth have erupted, the salivary ALP levels are also lower.

As previously described, ALP is synthesized and secreted by osteoblasts during bone formation, and also catalyzes the hydrolysis of ester phosphatase, which is an important inhibitor of mineralization processes in alkaline pH associated with the formation of calcified tissues. Increased osteoblast activity during bone formation will be accompanied by increased expression of the enzyme ALP. This enzyme activity is responsible for the calcification of collagen fibrils as the basic material of bone. The role of ALP in the process of bone mineralization is to prepare an alkaline atmosphere in the osteoid tissue formed, so that calcium can be easily deposited in the tissue. In addition, in bone this enzyme causes an increase in phosphate concentration, so that calcium phosphate bonds are formed in the form of hydroxyapatite crystals and based on the law of mass will eventually settle in the bone. 24

Osteoblasts originate from the bone marrow and are responsible for external matrix deposition and bone mineralization. Osteocytes are osteoblasts that are trapped in osteoid and become inactive. Osteoclasts are large, multinucleated phagocytic cells, and a derivative of monocytes or monocyte-like cells that originate from the bone marrow, these cells are responsible for bone resorption. Bone is continuously formed by osteoblasts and continuously resorbed by osteoclasts during the remodeling process. The interdependence of osteoblasts and osteoclasts is known as coupling. This process occurs in bone throughout the body, including the bone around the tooth seed that will prepare for tooth eruption. This is a strong reason how ALP levels increase significantly when more teeth erupt or in other words, the dental age is getting bigger. 25

### Gender Disparities in Dental Age and Salivary Biomarkers

This study found that dental age and salivary biomarkers consistently increase in all three age groups in female with higher result than in male. The observed gender disparities in dental age and salivary biomarkers may be attributed to biological and developmental differences between males and females, even in early childhood. Former studies have suggested that salivary protein and enzyme expression can vary by sex due to differences in endocrine regulation, growth velocity, and genetic factors influencing salivary gland function and reported distinct patterns of salivary protein composition between boys and girls, noting that sex-related differences are detectable even before puberty. 26 Similarly, Uher et al found that ALP activity in saliva exhibits sex-linked variation, possibly reflecting differences in bone turnover and mineralization processes, which are influenced by growth and early hormonal activity. 27

The notably high albumin levels in older females may also reflect physiological shifts associated with increased metabolic demand during periods of rapid growth. Growth spurts occur at slightly different timings in boys and girls during the first 2 years of life, leading to variation in salivary biomarker levels when stratified by age. In addition, local oral environmental factors such as salivary flow, oral microbial colonization, and mucosal permeability may differ subtly between genders, thereby influencing measurable salivary protein concentrations. 28

Although gender-related differences in salivary albumin and ALP levels were observed, the current evidence base on such disparities in early childhood remains limited. These findings should therefore be interpreted with caution, as the differences may also reflect the unequal distribution of participants across the three age groups rather than true biological variation.

### Study Limitations and Methodological Considerations

Several limitations should be acknowledged. First, the small sample size and single-center recruitment limit the generalizability of the findings. A convenience sampling approach was used, which may not capture the full variability of the target population. Second, this study is a cross-sectional study so it cannot describe the condition of albumin and ALP levels in saliva longitudinally. Third, the age range of 6 to 24 months was selected because the use of salivary biomarkers as a diagnostic tool is expected to be most beneficial in younger patients, where obtaining radiographic images is more challenging and may expose infants to unnecessary radiation. Saliva-based methods therefore offer a safer and more feasible alternative for this age group. Nevertheless, further studies involving older children are warranted to determine whether these biomarkers remain reliable indicators of dental age across wider developmental stages.

## Conclusion

This study demonstrates a significant relationship between salivary albumin and ALP levels and dental age in children aged 6 to 24 months, with both biomarkers showing an increasing trend alongside advancing dental age. These findings highlight the potential of salivary albumin and ALP as noninvasive indicators for predicting tooth eruption timing. However, while promising, these results require validation in larger and more diverse cohorts to strengthen their generalizability. Such studies would provide a clearer path for integrating salivary biomarkers into clinical practice for establishing reliable diagnostic tools.
